# A Mechanism for Reviewing Investments in Health Research Capacity Strengthening in Low- and Middle-Income Countries

**DOI:** 10.5334/aogh.2941

**Published:** 2020-08-03

**Authors:** Peter H. Kilmarx, Thabi Maitin, Taghreed Adam, Hannah Akuffo, Garry Aslanyan, Michael Cheetham, Rodrigo Corrêa-Oliveira, Simon Kay, Nadia Khelef, Yaso Kunaratnam, Linda Kupfer, Ole F. Olesen

**Affiliations:** 1 Fogarty International Center, National Institutes of Health, Bethesda, MD, US; 2 ESSENCE Working Group on Review of Investments, US; 3 South African Medical Research Council, Parow Valley, Cape Town, Western Cape, ZA; 4 Research for Health Department, World Health Organization, Geneva, CH; 5 Unit for Research Cooperation, Swedish International Development Agency, Sida, Stockholm, SE; 6 Special Programme for Research and Training in Tropical Diseases (TDR), World Health Organization, Geneva, CH; 7 Fundação Oswaldo Cruz (Fiocruz), Rio de Janeiro, BR; 8 Wellcome Trust, London, UK; 9 Institut Pasteur, Paris, FR; 10 UK Collaborative on Development Research, London, UK; 11 European & Developing Countries Clinical Trials Partnership (EDCTP), The Hague, NL

## Abstract

More than 40 agencies that fund health research capacity strengthening in low- and middle-income countries (LMICs) participate in the ESSENCE Health Research initiative, which has established a mechanism for reviewing and coordinating their funding. Taken together, the expected outcomes of implementation of the review mechanism are increases in the efficiency and equity in health research capacity strengthening activities with decreased duplication of efforts. The overall goal is increased support of research on national health priorities as well as improved pandemic preparedness in LMICs, and, eventually, fewer countries with very limited research capacity.

Research and innovation are needed to develop and implement new tools and interventions in order to achieve the health targets called for in the Sustainable Development Goals [1]. However, the existing capacity to conduct relevant research for health is insufficient, especially in low- and middle-income countries. Focusing on one important area, *Money and Microbes*, the World Bank’s *International Vaccine Task Force* 2018 report, concluded that clinical research capacity is inadequate in many LMIC settings where epidemic disease outbreaks often strike and recommended that health research capacity be considered a critical element of pandemic preparedness [2].

Multiple partners are engaged in efforts to strengthen health research capacity in LMIC, but currently, there is no global system for reviewing investments and coordinating these efforts. The *Task Force* recommended that the agencies in the *ESSENCE on Health Research*, which brings together funders of health research in LMICs, in collaboration with key stakeholders, articulate such a mechanism [2]. This *Editorial* describes the methods, main findings, and recommendations for the development and initial implementation of the review and coordination mechanism. A recent report commissioned by the UK Department for International Development also noted the lack of system-level data and coordination in health research capacity strengthening [3], while others have highlighted the need for external funders to collect, analyze, and report on their support for strengthening postgraduate health research capacity [4].

More than 40 agencies that fund health research capacity strengthening in LMICs are involved in *ESSENCE*, [5], which has its secretariat at the Special Programme for Research and Training in Tropical Diseases (TDR) at the World Health Organization (WHO), Geneva. To develop the review mechanism, *ESSENCE* members, with input from the *Global Coordination Mechanism for R&D Preparedness* [6] and LMIC representatives, convened a working group, and engaged a consultant who conducted a literature review and key informant interviews [7]. A consultation with about 40 diverse stakeholders involved in health research capacity strengthening in LMICs, including representatives from international scientific academies, research and development organizations, and other funding organizations from Africa, Asia, and Latin America [8].

The main findings of the background research and stakeholders meeting were as follows:

1. Establishment of a mechanism for reviewing investments in capacity strengthening for health research in LMICs would provide a common set of principles, metrics, data, and standards to better inform investment decisions. This would encourage greater synergy and enhanced coordination of funders of health research, research organizations, users of clinical research, and other key actors and improve stakeholder engagement.
2. The main barriers to setting priorities and coordinating health research capacity strengthening activities include the lack of a) quality data on what health research and capacity-strengthening programs are being conducted or planned, b) a shared set of metrics and quality data on current capacities at institutional and national levels, and c) a forum where sharing of information and coordination can take place.
3. Data-sharing systems should be sufficiently comprehensive to meet stakeholders’ needs but not too complex to be efficiently and cost-effectively implemented, maintained, and kept relevant.
4. The organizational entity or entities for the review process should maintain an LMIC focus with representation from LMICs, organizational agility and responsiveness, and neutrality, achieving collective accountability in a way that is non-threatening yet effective.

The mechanism for review of investments developed by the working group and endorsed by *ESSENCE* includes three interdependent workstreams (Figure 1):

1. The WHO Global Observatory on Health Research and Development (R&D) is establishing, in collaboration with WHO regional offices and member state representatives, a set of core metrics to characterize the status of health research capacity at the institutional and national levels in a standardized way worldwide [9]. In the interim, existing data from grants on health research, clinical trial registries, distribution of health researchers, and bibliometric analysis may be used to infer health research capacity levels.
2. World RePORT [10], an open-access, interactive database project that maps biomedical research investments and partnerships from some of the world’s largest funding organizations of health research, which should be strengthened and serve as a key resource.
3. *ESSENCE* itself will periodically convene global funders and stakeholders to review investments, identify gaps, and enable funders to enhance coordination and collaboration on these investments.

**Figure 1:**
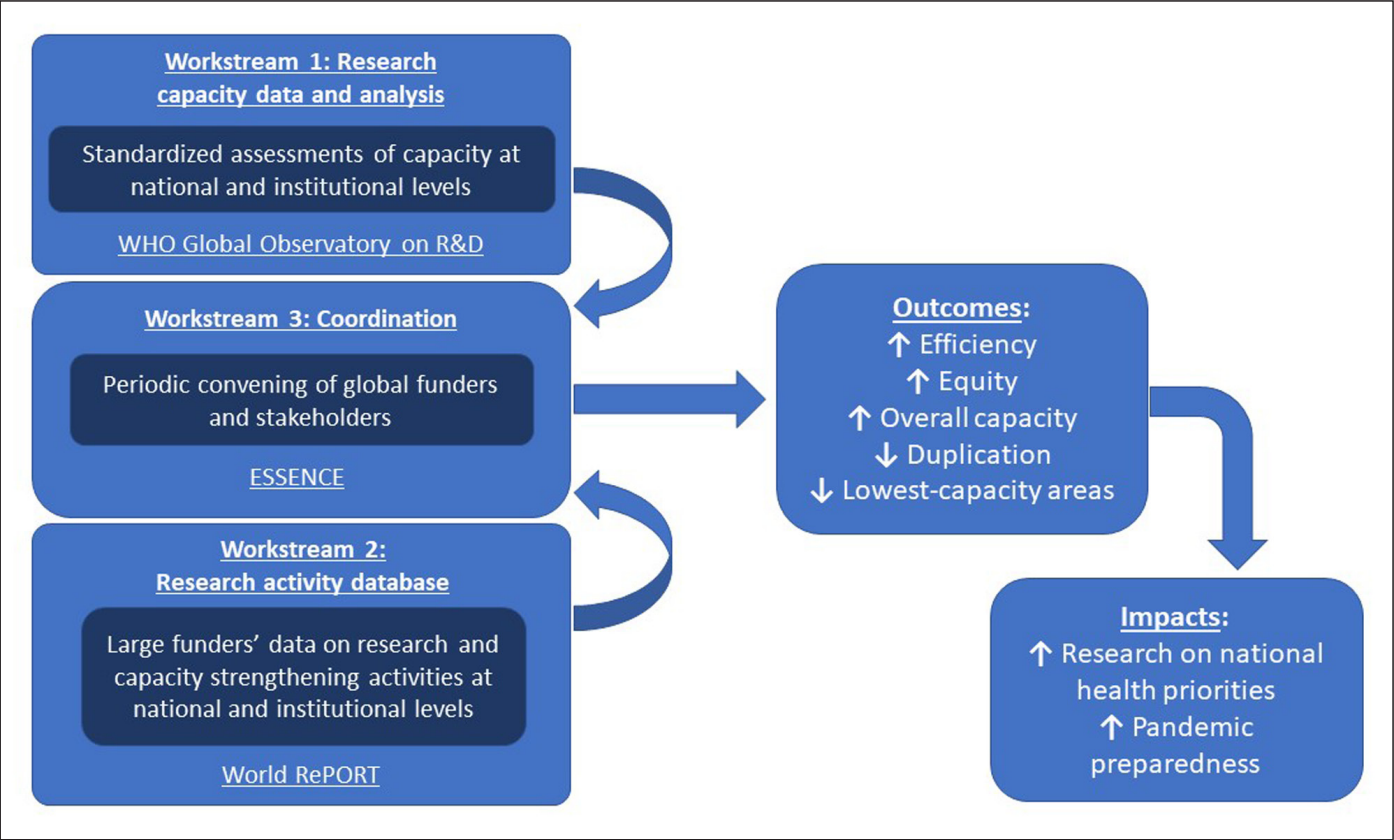
Schematic of Mechanism for Review of Investments in Health Research Capacity Strengthening in Low- and Middle-Income Countries.

These plans are relevant to the Global Pandemic Monitoring Board, which is tracking progress on global preparedness for health crises under the auspices of the UN General Assembly [11].

A critical next step will be the development of metrics for health research capacity, which is being led by the WHO Global Observatory [9]. The opportunity to establish and share harmonized and agreed-upon indicators should be expanded with additional resources to help ensure its sustainability. Many global health and development programs have specific indicators and targets used to establish road maps and budgets to mobilize resources within a framework for partner coordination. This is largely lacking for health research capacity strengthening efforts.

Standardized metrics and a common dataset on health research capacity across WHO regions will allow targeting of resources to institutions and countries at different capacity levels. At present, funding for health research capacity strengthening is often awarded competitively with eligibility limited to applicants in all LMICs or those in a specific region, e.g., sub-Saharan Africa. For some funders focused on research capacity strengthening in specific institutions, support is provided through a competitive process limited to the target institutions. Others support consortia, enabling the emergence of centers or networks of excellence that recruit fellows for training from countries with less capacity [12]. In the current system, highly competitive centers of excellence have emerged and competed successfully for multiple funding awards, while some institutions and countries with less health research capacity are left behind. In 2017, for example, 16 countries in Africa received about 90% of research grants from the funders in World RePORT, while 28 countries split the remaining 10%, and 11 countries received no grants. Furthermore, 24 African research institutions each received 25 or more grants, 57 research institutions received 10 or more grants, and 791 organizations received only one or two grants [13]. A robust system for characterizing health research capacity would allow targeting of some subset of resources for which only sites with less capacity would be eligible. This could lead to a more equitable distribution of resources, providing additional resources where needed, and helping establish at least a minimum capacity to conduct research to address national health priorities and for outbreak preparedness.

A validated system for characterizing health research capacity would further be helpful for developing and evaluating capacity-strengthening interventions. The inputs and support needed for institutions and countries with low levels of health research capacity are different from those needed at higher levels. Common metrics are needed to establish a framework for targeting support appropriately.

World RePORT currently includes data from 11 of the largest public and philanthropic funders of global health research [10]. It shows “who is doing what where.” It is useful, for example, to see what research is being supported by these funders in specific countries and regions, or to see where research supported by these funders on specific health conditions is being conducted. Planned enhancements include adding data from more funding organizations, more timely availability of data, and a more robust data platform for data search and analysis. World RePORT does not currently differentiate funding for health research capacity strengthening from funding for health research. Of note, much health research capacity strengthening takes place as an integral element of health research and not always as a specific separate aim. Ideally, future versions of World RePORT will specifically identify activities to strengthen research capacity in grants and will allow for forward-looking projections in case of multiyear awards.

ESSENCE is planning the first of the periodic consultations 2020, with LMIC representatives and other key stakeholders to review the available data on current health research capacity and capacity-strengthening activities. Discussions will focus on the potential to increase coordination of health research capacity-strengthening efforts and address geographic gaps in a health research capacity, reduce duplication, increase efficiency, and optimize the equitable distribution of resources. Best practices and lessons learned will be shared on strategies to strengthen health research capacity and the experience with supporting institutions and countries with limited levels of health research capacity. Such a data-driven forum focused on strengthening capacity for health research will be critical to accelerate progress. However, challenges to increasing coordination may be anticipated in light of the very broad spectrum of the types and missions of each funding organization, as well as the diverse categories of health-related research supported.

Taken together, the expected outcomes of the implementation of the review mechanism are increases in the efficiency and equity in health research capacity strengthening activities with decreased duplication of efforts. The overall goal will be increased support of research on national health priorities as well as improved pandemic preparedness in LMICs, and, eventually, fewer countries with very limited research capacity.
